# Efficacy and Cost-Effective Treatment of Isometric Resistance Training for Blood Pressure Control: A Narrative Review in Healthy Individuals and People with Cardiovascular Diseases

**DOI:** 10.3390/jfmk11030316

**Published:** 2026-08-13

**Authors:** Matteo Vitarelli, Domenico Mario Giamundo, Alberto Grossi, Giuseppe Caminiti, Gabriele Morganti, Francesca Strassoldo di Villanova, Bruno Ruscello, Elvira Padua, Maurizio Volterrani, Valentina Morsella, Marco Alfonso Perrone, Vincenzo Manzi, Gennaro De Rosa, Lorenzo Loffredo, Enrico Maggio

**Affiliations:** 1 Department of Human Science and Promotion of Quality of Life, San Raffaele Open University, 00166 Rome, Italy; alberto.grossi@uniroma5.it (A.G.); gabriele.morganti@uniroma5.it (G.M.); francesca.strassoldo@uniroma5.it (F.S.d.V.); bruno.ruscello@uniroma5.it (B.R.); elvira.padua@uniroma5.it (E.P.); maurizio.volterrani@uniroma5.it (M.V.); 2 Department of Neurosciences, Biomedicine and Movement, University of Verona, 37134 Verona, Italy; 3 Division of Cardiology, Policlinico Casilino, 00133 Rome, Italy; jamundus20@libero.it; 4 IRCCS San Raffaele Roma, 00166 Rome, Italy; valentina.morsella@sanraffaele.it; 5 Department of Industrial Engineering, Faculty of Engineering, “Tor Vergata” University, 00133 Rome, Italy; 6 LUISS SportLab, LUISS University, 00197 Rome, Italy; 7 Division of Cardiology and Sports Medicine, Department of Clinical Sciences and Translational Medicine, University of Rome Tor Vergata, 00133 Rome, Italy; marco.perrone@uniroma2.it; 8 Department of Wellbeing, Nutrition and Sport, Pegaso Open University, 80143 Naples, Italy; vincenzo.manzi@unipegaso.it; 9 Department of Advanced Biomedical Sciences, Division of Cardiology, University of Naples Federico II, 80131 Naples, Italy; gennaro.derosa3@unina.it; 10 Interdisciplinary Department of Well-Being, Health and Environmental Sustainability (BESSA), Sapienza University of Rome, 02100 Rieti, Italy; lorenzo.loffredo@uniroma1.it; 11 Department of Medical and Cardiovascular Sciences, Sapienza University of Rome, Viale del Policlinico 155, 00161 Rome, Italy; enrico.maggio@uniroma1.it

**Keywords:** isometric exercise, hypertension, cardiovascular diseases

## Abstract

Isometric resistance training (IRT) has emerged as a promising non-pharmacological intervention for blood pressure (BP) management. Although current hypertension guidelines primarily recommend aerobic and dynamic resistance exercise, growing evidence indicates that IRT is an effective, feasible, and time-efficient alternative. This narrative review summarizes the current evidence on the efficacy, physiological mechanisms, and clinical applications of IRT. We reviewed randomized controlled trials, systematic reviews, and meta-analyses evaluating the effects of different IRT modalities, including isometric handgrip (IHG), isometric wall squat (IWS), and isometric leg extension (ILE), on BP in normotensive individuals, patients with hypertension, and those with cardiovascular disease. IRT consistently reduces resting systolic BP by approximately 5–8 mmHg and diastolic BP by 3–4 mmHg; these effects may approach those reported with first-line antihypertensive therapy and potentially be equivalent to those of other exercise modalities. These benefits have been demonstrated across normotensive and hypertensive populations and appear largely independent of age, sex, medication use, and the muscle groups involved. Preliminary evidence also supports the feasibility and safety of appropriately prescribed IRT in selected patients with ischemic heart disease and heart failure with preserved ejection fraction. Proposed mechanisms include improvements in endothelial function, nitric oxide bioavailability, autonomic regulation, oxidative stress, arterial baroreflex sensitivity, and myocardial efficiency. IRT has emerged as an effective, safe, and practical adjunct to lifestyle modification for BP control, particularly in older adults and individuals with limited ability to perform conventional exercise. However, current evidence derives from relatively small studies with limited follow-up; further large-scale randomized trials are needed to define the optimal exercise prescription, clarify the underlying mechanisms, and establish the role of IRT in patients with cardiovascular disease.

## 1. Introduction

Hypertension remains one of the most prevalent and impactful modifiable cardiovascular risk factors worldwide, contributing substantially to global morbidity and mortality through its association with ischemic heart disease (IHD), stroke, heart failure, and chronic kidney disease [1,2]. Despite advances in pharmacological therapy, optimal blood pressure (BP) control is frequently not achieved, with a significant proportion of patients remaining above recommended targets even when treated with multiple antihypertensive agents [2,3]. This persistent therapeutic gap has reinforced the importance of non-pharmacological strategies, particularly lifestyle interventions, in both the prevention and management of elevated BP values. Among these, regular physical activity (PA) is widely recognized as a cornerstone intervention, with consistent evidence demonstrating clinically meaningful reductions in both systolic and diastolic BP [4,5]. Current international guidelines predominantly recommend the combination of aerobic and dynamic resistance training as a first-line approach [3,6]. However, adherence to these recommendations remains suboptimal, often limited by time constraints, physical deconditioning, comorbidities, or poor tolerability in certain populations, including older individuals and patients with cardiovascular disease [7]. These limitations have prompted growing interest in alternative exercise modalities that are both effective and more easily implementable in clinical practice. Isometric resistance training (IRT) has emerged as a promising and practical intervention in this context. Characterized by sustained muscular contraction without changes in muscle length, IRT is typically performed using brief bouts of exercise at different intensities, interspersed with rest periods, and can be delivered using simple, low-cost equipment in both supervised and home-based settings [8]. Its feasibility, minimal time requirement, and ease of integration into daily routines make it particularly attractive for individuals who may struggle to adhere to traditional exercise prescriptions. Nevertheless, in chronic or macrocycle training, the hypotensive benefits may depend on maintaining adherence and ensuring adequate supervision, particularly during the initial phases of training due to familiarization with the exercises. Over the past decade, a growing body of evidence has consistently demonstrated that IRT induces significant reductions in resting BP. Meta-analytic data indicate average decreases of approximately 7–8 mmHg in systolic and 3–4 mmHg in diastolic BP, magnitudes that are comparable to those observed with single-agent antihypertensive therapy [9,10,11]. Importantly, these reductions are clinically meaningful, as even modest decreases in BP have been associated with substantial reductions in cardiovascular events, including stroke and myocardial infarction [12]. Furthermore, recent large-scale network meta-analyses have positioned IRT as the most effective exercise modality for BP reduction when compared with aerobic, dynamic resistance, and combined exercise interventions [5]. The antihypertensive effects of IRT appear to extend beyond hypertensive populations, with evidence also supporting its role in normotensive individuals, suggesting potential utility in primary prevention [5,13]. Despite the accumulating evidence supporting its efficacy and safety, IRT remains underutilized in clinical practice and is not yet fully integrated into standard hypertension management guidelines [5,9]. Taken together, these considerations highlight both the potential and the current limitations of isometric exercise (IE) as a therapeutic strategy for BP control. The aim of this narrative review is to critically examine the current evidence on the effects of IRT on BP, with particular emphasis on its role in normotensive individuals, patients with hypertension, and those with cardiovascular disease. Unlike previous reviews and meta-analyses, which have predominantly focused on healthy or hypertensive populations, this review extends the discussion to patients with established cardiovascular disease by synthesizing the available evidence on efficacy, safety, underlying physiological mechanisms, and potential clinical implications of incorporating IRT into cardiovascular prevention and rehabilitation.

## 2. Literature Search Strategy

Given the narrative nature of this review, a literature search was conducted between March and the end of June 2026 to identify the main evidence regarding the effects and clinical applications of IRT for BP control. PubMed and Scopus served as primary bibliographic databases to identify relevant publications, using combinations of terms related to isometric exercise, isometric resistance training, isometric handgrip, isometric leg exercise, wall squat, blood pressure, hypertension, and cardiovascular disease. The search strategy was complemented by a snowball approach, consisting of backward tracing citations of relevant articles and reviews and targeted identification of subsequent publications citing key studies. Additional studies were identified based on the authors’ expertise in exercise physiology and cardiovascular rehabilitation, with selection guided by their relevance to the specific clinical and mechanistic topics covered in the review. No formal risk of bias assessment or quantitative synthesis was performed, as the aim of this review was to provide a clinically oriented narrative synthesis of the available evidence rather than a systematic evaluation of treatment effects. The language filter was English.

**Inclusion criteria:**

Studies were considered eligible when they investigated defined IE and reported outcomes relevant to BP, cardiovascular function, physiological mechanisms, safety, feasibility, adherence or clinical applicability. RCTs, prospective interventional cohort studies, systematic reviews, meta-analyses and crossover studies were considered. Particular attention was given to studies conducted in normotensive or hypertensive adults and in populations with established cardiovascular disease, including IHD.

**Exclusion criteria:**

Studies were excluded when they did not investigate isometric exercise as the primary intervention or did not provide relevant cardiovascular or BP-related outcomes.

## 3. Cardiovascular Effects of Isometric Exercise

Mechanisms underlying the cardiovascular benefits of IRT are complex and likely multifactorial. Proposed pathways include improvements in endothelial function and nitric oxide (NO) bioavailability, enhanced vasodilatory capacity induced by repeated ischemia–reperfusion stimuli, reductions in oxidative stress, and favourable modulation of autonomic nervous system activity and arterial baroreflex sensitivity [14]. Notably, evidence linking oxidative stress to the antihypertensive effects of IRT is largely based on relatively small studies, which justifies that the evidence remains limited. The static and sustained nature of isometric contractions leads to elevated intramuscular pressures, which may compress intramuscular blood vessels and consequently reduce or even temporarily obstruct blood flow to the contracting muscle, resulting in transient ischemia [15]. During the subsequent relaxation phase, reperfusion occurs and is accompanied by reactive hyperemia. This response is facilitated by vasodilation, enhances muscle oxygen delivery, and may improve endothelial function, thereby mimicking the physiological effects of repeated ischemia–reperfusion stimuli (Khor). These vascular adaptations have been proposed as a potential mechanism underlying the sustained reduction in BP observed for several hours following an IE session [16]. A recent meta-analysis [17] of 23 studies evaluated the effects of resistance exercise, including both isometric and dynamic modalities, on flow-mediated dilation (FMD), a widely recognized marker of endothelial function. Notably, the analysis included a broad spectrum of populations, ranging from healthy individuals to patients with cardiovascular and metabolic disorders. However, only seven of 23 studies examined IRT, and all used isometric handgrip (IHG) as an exercise modality. Among these, four studies were conducted in healthy participants, two in patients with hypertension, and only one in individuals with peripheral artery disease. Consequently, the current evidence supporting the beneficial effects of IRT on endothelial-dependent vasodilation remains limited, particularly in patients with cardiovascular disease. Furthermore, there is a substantial lack of data regarding the vascular effects of IRT modalities other than IHG, highlighting the need for further investigation. The improvement in endothelial function associated with IRT may also be mediated by favourable changes in oxidative stress. The first study examining the effects of IHG training on oxidative stress biomarkers demonstrated that six weeks of training improved the ratio of reduced to oxidized glutathione, indicating a reduction in systemic oxidative stress [18]. However, the absence of a control group limited the strength of these findings. More recently, Olher et al. [19] reported that a single bout of IE involving a large muscle mass elicited a transient increase in pro-oxidant activity, which was accompanied by enhanced NO bioavailability and a compensatory upregulation of antioxidant systems. These findings suggest that redox-sensitive mechanisms may contribute to the vascular adaptations and BP-lowering effects associated with IRT. Evidence regarding the effects of IRT on autonomic nervous system regulation is similarly limited, and available results are contradictory. Most studies have assessed sympathovagal balance through changes in heart rate variability (HRV). An increase in high-frequency (HF) power, a recognized marker of cardiac vagal modulation following a 10-week IRT intervention, was reported by Taylor et al. [20]. Conversely, other investigations failed to demonstrate significant changes in HRV indices following IRT interventions lasting 4–10 weeks [21,22,23]. Dempster et al. [24] reported an increase in arterial baroreflex sensitivity after eight weeks of IHG training in treated hypertensive patients. More recently, an acute improvement in baroreflex sensitivity, following a single session of IRT, has also been observed in patients with chronic kidney disease [25]. Preliminary results indicate that BP reduction elicited by IRT can generate central hemodynamic benefits in the post-exercise phase. In an acute setting, O’Driscoll et al. [26] observed significant improvement in several metrics of left ventricular function, including ejection fraction and global longitudinal strain, after a single bout of IRT. The same group observed that global longitudinal strain (−2.3 ± 2%) and global work efficiency (2.8 ± 2%) significantly improved in 24 unmedicated hypertensive patients following four weeks of isometric wall squat (IWS) [26]. Overall, currently available evidence [27] suggests that autonomic and vascular adaptations may contribute to the BP-lowering effects of IRT. However, the relative contribution of endothelial function, oxidative stress modulation, and autonomic regulation remains incompletely understood, particularly in patients with cardiovascular disease. Further mechanistic studies are warranted to clarify these pathways.

## 4. IRT in Normotensive Healthy Subjects

Different IRT modalities have shown BP-lowering effects in normotensive healthy subjects [28,29,30,31,32,33]. Collectively, these studies have sought to quantify the magnitude of IRT’s effect on BP compared with other exercise modalities, identify the exercise intensity associated with the greatest BP reduction, determine the time course of the BP–lowering response, and investigate potential sex-related differences in the BP response to IRT. A randomized controlled trial (RCT) published by Goessler et al. [34] compared home-based IHG exercise versus endurance training or control in sixty healthy individuals (29 women and 31 men; 33.1 ± 1.3 years; 172.1 ± 1.0 cm; 78.1 ± 2.0 kg). Both exercise interventions were performed in the home environment. The IHG group performed four sessions of two minutes at 30% of maximal voluntary contraction (MVC) each day, while participants randomized to the aerobic group performed at least 150 min of continuous moderate-intensity exercise weekly. Compared with the control group, both aerobic and IHG reduced office BP, while only aerobic exercise reduced ambulatory BP. Wiles et al. [35] compared the effects of bilateral isometric leg extension (ILE), performed at two different intensities, on resting BP. Thirty-three male participants (30 ± 7 years; 178 ± 5 cm; 78.7 ± 11.1 kg) were randomized to high-intensity training, low-intensity training, or inactive control for eight weeks. Participants of the two active groups performed four sessions of two-minute exercise three times a week. Resting BP was measured after four and eight weeks of training. The study showed a reduction in systolic BP, diastolic BP, and mean arterial pressure (MAP) in both groups after eight weeks of training. Conversely, there were no significant changes in BP in both groups in four weeks. These results suggest that the BP-lowering effects of ILE are already observed at low intensity but, in order to obtain consistent results, at least 8 weeks of training are needed. Nevertheless, it is important to note that, given the small sample size of the study, these results should be interpreted with caution. In another study, the same authors [36] investigated the efficacy of a home-based IWS training in reducing resting BP. Twenty-eight healthy normotensive males (aged between 18 and 34 years; 179.4 ± 7.6 cm; 75.6 ± 12.2 kg) were randomly allocated to IWS or control. The interventional protocol included four weeks of home-based exercise using a crossover design with four weeks of rest (“washout period”) in between. IWS was performed in sessions of four repetitions of two minutes three times a week for 4 weeks, with 48 h of rest between each session. Interestingly, each session of IWS was performed at a participant-specific knee joint angle in order to obtain a target HR of 95% of HR peak, with two minutes of rest between each bout. This study showed significant reductions in BP and HR in the IWS group. The authors hypothesized that this BP-lowering effect observed was driven by the reduction of cardiac output secondary to the decrease in HR, since no significant between-groups changes in total peripheral resistance and stroke volume were observed, suggesting that the effect on BP of IWS was predominantly attributable to cardiac rather than peripheral vascular adaptations. Again, the small sample size limits the significance of these results, which should be confirmed in larger cohorts. Hess et al. [37] performed an RCT aimed to identify the minimum threshold intensity for IRT to have an antihypertensive effect. The study enrolled twenty-two normotensive subjects (12 males and 8 women completed the study; 38.8 ± 11 years; 174 ± 10 cm; 84.3 ± 24.2 kg) who were randomly allocated to IHG at either 5% or 10% of their MVC. Clinically meaningful, but not statistically significant, reductions in systolic BP were observed in both 5% and 10% groups after six weeks of training, with no diastolic BP reduction in either group. The failure to achieve statistical significance in this study is attributable, at least in part, to the small number of patients enrolled. Baross et al. [38] evaluated IRT in healthy middle-aged to older (45–60 years) men. Twenty male participants (55 ± 6 years; 181.2 ± 4.4 cm; 89.6 ± 3.1 kg) were randomized to a training or control group. The training group performed three ILE sessions each week for 8 weeks, at 85% of HR peak. The training resulted in a statistically and clinically significant reduction in resting systolic BP and MAP, with no significant changes in diastolic BP. Unfortunately, despite the interesting results, they must be interpreted in light of the small sample size. A prospective study by Badrov et al. [39] analyzed the presence of sex differences in the response to IHG. Resting BP and endothelium-dependent vasodilation, assessed through the evaluation of brachial artery FMD, were assessed in eleven young women (23 ± 4 years; 165 ± 5 cm; 58 ± 6 kg) and nine young men (21 ± 2 years; 179 ± 8 cm; 77 ± 12 kg) (overall population: 22.1 ± 3.3 years; 171.3 ± 9.5 cm; 66.6 ± 13.2 kg) at baseline and after eight weeks of IRT. Subjects performed four sessions of two minutes with unilateral contractions at 30% MVC, repeated three days a week. The study showed that IHG reduced systolic and diastolic BP, MAP, and pulse pressure in a clinically and statistically significant manner. Moreover, there was a statistically significant increase in absolute and relative brachial artery FMD. There were no significant between-gender differences in any of the aforementioned variables. Similarly to some of the cited studies, this study is also limited by the number of patients enrolled. The meta-analysis of Smart et al. [10] synthesized results of 12 RCTs on both normotensive and hypertensive patients, considering a total of 326 patients. Of these 326 patients, 52.7% received medication for hypertension; 191 patients were assigned to IRT, while the other 135 were allocated to controls. Among the various analyzed studies, training duration ranged from three to twelve weeks, while IRT intensity varied between 8 and 30% of MVC. Interestingly, this meta-analysis provided several important insights. First, it found no evidence that the BP response to IRT was influenced by either sex or age. Second, the magnitude of BP reduction was comparable in normotensive and hypertensive individuals, indicating that the antihypertensive effects of IRT are not restricted to patients with elevated baseline BP. Likewise, the presence of antihypertensive medication did not significantly modify the BP response to IRT. Finally, no significant differences in treatment effect were observed between protocols involving upper- and lower-limb IRT, suggesting that the antihypertensive benefits of IRT are largely independent of the muscle groups engaged. A subsequent meta-analysis [40] specifically evaluating normotensive individuals included six RCTs, three employing ILE at different exercise intensities, two using IHG at 30% of maximal voluntary contraction (MVC), and one using isometric arm flexion. Overall, IRT was associated with significant reductions in systolic BP (−2.83 mmHg), diastolic BP (−2.73 mmHg), and MAP (−3.0 mmHg), confirming that this training modality exerts a modest but clinically relevant antihypertensive effect even in individuals with normal resting BP. There are few direct comparisons among different IRT modalities regarding their effects on BP. In an elegant cross-over study, Edwards et al. [41] compared the effects of IWS versus IHG at 30% MVC. The authors randomized 21 healthy subjects (12 women and 9 men; 33 ± 14.1 years; 171 ± 10.3 cm; 76 ± 12.7 kg) to a 4-week IWS or IHG intervention, followed by a 4-week ‘washout’ period before crossing over to the alternate IRT modality. Although both IRT modalities obtained BP reductions, IWS produced significantly greater decreases in MAP (−2.3 ± 0.8 mmHg) and diastolic BP (−2.4 ± 1.1 mmHg) compared with IHG, suggesting a superior antihypertensive effect of large-muscle-group IE. Also in this case, the sample size should be counted among the study’s limitations. The acute response to a single bout of IRT, defined as post-exercise hypotension (PEH), has also been investigated in healthy young individuals: in a two-center study, Somani et al. [42] evaluated the acute response of 46 healthy young individuals (23 women and 23 men; 24 ± 5 years; 171 ± 12 cm; 72.5 ± 14.5 kg) to different forms of IRT to predict the PEH induced by these different types of IRT. Resting BP and PEH to IRT were assessed at baseline and after ten weeks of exercise, performed three times a week. The Canadian participants performed IHG, while participants in the UK performed ILE. There was a statistically significant reduction in both groups in systolic BP and pulse pressure. Moreover, PEH induced by IHG and ILE were associated with the changes in systolic BP induced by the IRT training (r = 0.58 for IHG and r = 0.77 for ILE). These results suggested that acute BP response to an IRT could be a useful tool to identify patients who may respond to long-term IRT prescription (Table 1). In light of what has been discussed in this chapter, it is important to underline that the different analyzed studies have a relevant methodological heterogeneity in terms of exercise intensity, programme duration and medication use.

## 5. IRT in Hypertensive Patients

Different studies have investigated the role of IRT in patients with hypertension. Carlson et al. [43] investigated forty middle-aged patients with pre-hypertension or mild hypertension, performing IRT for 8 weeks. Participants were randomized into a low-intensity group (5% MVC) or into a high-intensity group (30% MVC). The protocol used consisted of four series of two-minute IHG exercises with a three-minute rest period between each series, with a frequency of three days a week. After eight weeks of training, the 30%-MVC group experienced a significant drop in systolic BP (−7 mmHg) and MAP (−4 mmHg), while no statistically significant changes in systolic BP or MAP occurred in the 5% MVC group. Similar results were obtained by Palmeira et al. [44], who analyzed the effects of IRT on office and ambulatory BP in 63 hypertensive patients (70% of whom were female). Participants underwent a twelve-week training protocol consisting of four series of two-minute IHG at 30% MVC with 1-min rest between bouts. That session was repeated three times a week; the authors observed a statistically significant reduction in office systolic BP (−8 mmHg), but no effect on diastolic BP, HRV, or ambulatory BP. An interesting trial, published in 2018 [45], randomly assigned 72 hypertensive patients to three groups: home-based IRT, supervised IRT, or control groups. The exercise protocol consisted of four series of two minutes at 30%-MVC, with one-minute rest between bouts, repeated three times a week. At 12 weeks, the authors observed a significant reduction in brachial systolic BP by 12 mmHg and in brachial diastolic BP by 5 mmHg in the supervised group; conversely, no significant changes in brachial systolic BP and diastolic BP were observed in the home-based group. Furthermore, supervised handgrip exercise also significantly reduced central systolic BP by 11 mmHg, diastolic BP by 6 mmHg and MAP by 9 mmHg. Again, no changes were observed in systolic BP, diastolic BP and MAP in the home-based group. There were no significant effects on ambulatory BP, arterial stiffness, HRV, vascular function, oxidative stress and inflammatory markers in all three groups. The results of this study clearly underscored the superiority of supervised training compared to a home-based intervention for lowering BP in hypertensive patients. A systematic review and meta-analysis focusing on the use of IRT for the management of hypertension summarized 12 RCTs accounting for 415 participants. Interestingly, in 11 of the 12 studies included in the analysis, IHG was the modality of IRT employed, highlighting the predominant use of this exercise model in the available literature. Results showed that IRT reduced SBP by 7.47 mmHg, DBP by 3.17 mmHg, and MAP by 7.19 mmHg [8]. Subgroup analyses according to medication status showed that IRT-induced reductions in systolic BP were attenuated in medicated participants (−6.1 mmHg) compared with unmedicated individuals (−12.8 mmHg). Nevertheless, BP reductions remained statistically significant in both groups (*p* < 0.001). Notably, all studies included in this meta-analysis employed relatively short-term interventions, with IRT protocols lasting between 4 and 8 weeks, thereby limiting conclusions regarding the long-term efficacy of this training modality. To overcome this limitation, the meta-analysis of Yuan et al. [46] selectively included RCTs with training intervention duration ≥ 8 weeks. In this case, the pooled evidence showed only modest and no statistically significant changes in both systolic BP (−2.3 mmHg) and diastolic BP (−1.3 mmHg). It’s important to underline that this meta-analysis was limited by the relatively small sample size (112 patients in the intervention group and 116 in the control group) and by the moderate to high risk of bias of the studies considered. For example, none of the studies described specific strategies used to maintain participant adherence; only 2 studies explicitly reported the method for quantifying adherence, and only one study reported the deviation rate between actual training intensity and the planned intensity. Previous studies evaluating PA levels and adherence to exercise recommendations in patients with cardiovascular disease have consistently reported suboptimal long-term adherence, with fewer than 50% of participants maintaining regular exercise at 12 months [47]. Evidence regarding adherence specifically to IRT remains limited because of the small sample size and relatively short follow-up period of studies. Therefore, whether adherence to IRT is comparable to that observed with other exercise modalities remains to be established. Interestingly, in the feasibility trial by Wiles et al. [48], 34% of the 41 enrolled participants had withdrawn by the 6-month follow-up. Nevertheless, among participants who completed the intervention, 85% of the prescribed IRT sessions were performed at the target exercise intensity, indicating good adherence to the training protocol. Further evidence on the long-term feasibility and adherence to IRT is expected from the ongoing ISOFITTER trial, a large, randomized study evaluating the long-term effects of IRT in patients with stage 1 and stage 2 hypertension [49]. The results of this trial are anticipated to provide important insights into long-term treatment adherence, as well as the clinical effectiveness and sustainability of IRT in routine practice. All the aforementioned studies evaluated the chronic effects of IRT on hypertensive patients. The PEH effect evoked by a single bout of IRT in patients with hypertension has been poorly investigated and led to conflicting results. A small, randomized crossover trial by Silva et al. [50] analyzed 12 patients who underwent four IHG sessions in a random order: four sessions of two minutes at 30% of MVC; four sessions of two minutes at 50% of MVC; four sessions of three minutes at 30% of MVC; and a control session. There were no significant changes in systolic BP, diastolic BP, HR, and rate-pressure product between pre- and post-exercise (30th minute) measurements after any session for all comparisons. Similarly, individual analyses showed heterogeneity in the responses, including increases in BP observed in some sessions, and those patients with a reduction in BP had a higher body mass index, diastolic BP, and HR (*p* < 0.05). Another study, by Bocalini et al. [51], investigated the occurrence of PEH following different intensities of IHG in 12 hypertensive elderly women under antihypertensive medications. Patients underwent two sessions of IRT performed in four repetitions of five contractions of 10-s duration; the sessions were performed both at 30% and 50% of MVC. Both intensities were compared with a control session without exercise, and the measurements were performed at rest, during peak exercise, and for 60 min during the post-exercise phase. The authors observed no significant changes in systolic and diastolic BP after any session for all comparisons, with no episodes of hypotension. These results were not confirmed by other studies that showed significant PEH after single bouts of low-intensity IHG and ILE in patients with pre-hypertension/hypertension 1 [52] or hypertension [53]. The differing results reported by these latter studies may be attributable to the longer duration of post-exercise BP monitoring, which extended to 6 h in the study by van Assche et al. [52] and to 24 h in the study by Oliveira et al. [53]. To summarize some of the available evidence, a systematic review by Farah et al. [54] analyzed five studies on chronic and two studies on acute effects of IRT. The authors’ results showed that none of the acute studies reported PEH after a single session of IRT, and that most of the chronic studies, with training ranging from 6 to 10 weeks, showed a reduction in office BP. Clearly, it was not technically valid to perform a meta-analysis based on only two studies, and therefore the published results on the acute studies are intended as speculative and with the aim of stimulating further research. A recent umbrella review summarized the evidence regarding the effectiveness of IRT in patients with hypertension [55]. This research considered twelve systematic reviews with meta-analysis published between 2011 and 2021 of varying methodological quality; of them, IHG training with four series of two-minute contractions and a one-minute rest period between each series, repeated three times per week for at least eight weeks, was the most used protocol. The authors observed consistent evidence indicating IRT as an effective intervention to reduce systolic BP, diastolic BP, and MAP. The study included in this umbrella review considered both normotensive and hypertensive patients, and these positive impacts were reported for both types of individuals (Table 2).

## 6. IRT in Hypertensive Patients with Cardiovascular Diseases

### 6.1. Ischemic Heart Disease

**Figure 1:**
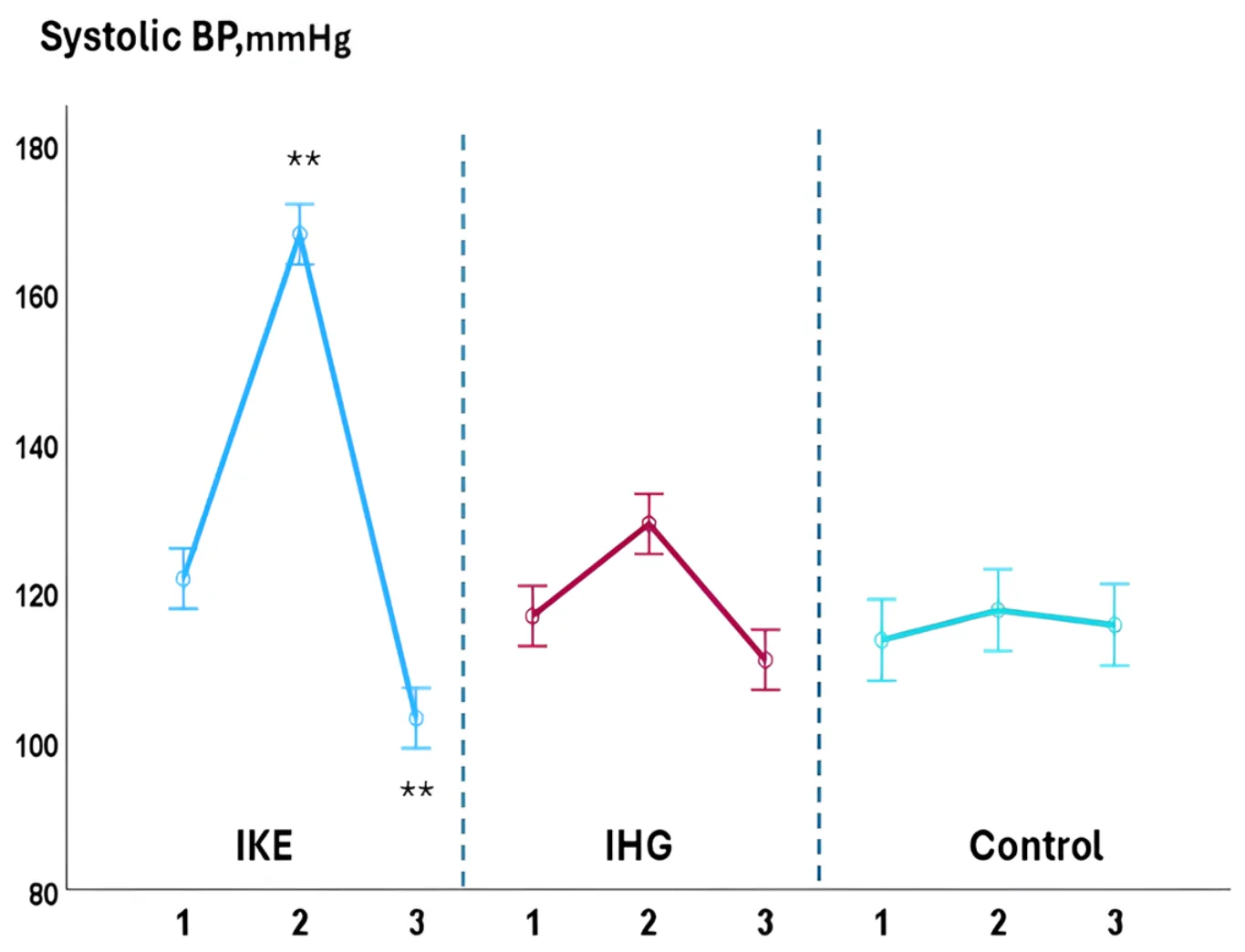
Changes in systolic BP during three phases of exercise (1 = baseline; 2 = exercise; 3 = recovery) among the three groups (isometric knee extension [IKE], isometric handgrip [IHG] and controls) analyzed in the study of Caminiti et al. [61]; ** = *p* < 0.05 versus the active group and control group.

Traditionally, IRT has not been incorporated into clinical practice, mainly due to historical concerns regarding excessive BP responses [56]. Although in 1987 Hanson and Nagle [57] stated that “*submaximum IE is well tolerated in cardiac patients with mild impairment of left ventricular function and may be used as part of a supervised exercise training program*”, IRT has not been implemented in cardiac patients since then and current guideline do not include IRT among suitable exercise modalities for cardiovascular patients since no trials are investigating the effects of IRT on patients with IHD. In the meta-analysis of Smart et al. [10], published in 2019, the authors underlined that there were insufficient people with coronary heart disease enrolled in RCTs to draw meaningful conclusions regarding the effects of IRT on BP in this group. Interest in using IRT in patients with cardiovascular disease has grown substantially in recent years for three main reasons. First, the accumulating evidence consistently demonstrates the efficacy of IRT in reducing BP in both normotensive and hypertensive individuals. Second, unlike aerobic exercise, IRT has the potential to improve skeletal muscle strength and, to a lesser extent, muscle mass while simultaneously lowering BP. This dual benefit is particularly relevant in older adults and patients with chronic cardiovascular disease, in whom sarcopenia and muscle weakness are common and contribute to reduced functional capacity and exercise tolerance [58]. Third, the ability of IRT to elicit clinically meaningful reductions in BP through simple, time-efficient exercise protocols, typically requiring sessions of approximately 20 min, makes it an attractive and potentially more acceptable exercise modality for older individuals and for patients with limited time or poor adherence to conventional exercise programmes. Finally, preliminary findings suggest that 12 weeks of IRT may stimulate angiogenesis by increasing vascular endothelial growth factor (VEGF) levels, thereby promoting the development of coronary collateral circulation [59]. The safety of IE, performed in different modalities and intensities, in hypertensive patients with underlying IHD has been recently investigated in the acute setting, using speckle tracking echocardiography. Caminiti et al. [60] compared the hemodynamic impact of ILE, performed at 30% MVC, in twenty stable untrained hypertensive patients with IHD versus ten healthy, age-matched controls. Although the study demonstrated that a single bout of IE was clinically well tolerated by every subject, at peak exercise, systolic BP was significantly higher in patients with IHD compared to healthy controls (37.6 ± 7.2 vs. 8.4 ± 2.3 mmHg). Correspondingly, E/e’ increased and atrial strain decreased in the IHD group. More importantly, in IHD patients, global wasted work increased in a greater proportion compared to global constructive work, leading to a significant reduction of global work efficiency. According to the authors, these alterations may have impaired the expected increase in stroke volume during the isometric contraction phase. The same authors compared the acute hemodynamic impact of ILE versus IHG, both performed at 30% of MVC in trained IHD patients [61]. Again, they observed a significant increase in systolic BP in the ILE group coupled with an increase in wasted work and a significant reduction in global work efficiency as shown in Figure 1. It should be noted, however, that these changes in hemodynamic parameters were much less extensive than those observed in the previous study despite the intensity of ILE used being the same. According to the authors, the differences in the hemodynamic response to ILE between the two studies may be explained by differences in the participants’ training status. No changes in BP and echocardiography parameters were observed during IHG, suggesting that low-intensity IHG has neutral hemodynamic effects on IHD patients during the contraction phase [62]. The effectiveness of IE in reducing BP in IHD patients on top of therapy has been poorly investigated. Available data have almost exclusively been obtained in acute settings. In a recent pilot study, Vitarelli et al. [63] compared the PEH effect of three different sessions: continuous moderate-intensity aerobic exercise, bilateral leg extension (BLE) performed at 20% of MVC, and no-exercise. They found that both aerobic and BLE sessions elicited a significant systolic PEH compared to no-exercise. However, the aerobic session was associated with the greatest PEH response. It should be noted, however, that the durations of the exercise sessions differed, with the isometric session lasting 20 min and the aerobic session lasting 60 min. A recent study comparing low (30% MVC) versus high (70% MVC) intensity IHG found that the high-intensity elicited greater PEH effects than the low-intensity. While there were no significant differences between IHG-70% and IHG-30% at different time points, systolic BP decreased significantly in IHG-70% compared to control [62]. Interestingly, both intensities had neutral effects on hemodynamic parameters. These results are consistent with Gordon et al. [64], who performed a small pilot study on IHG patients undergoing cardiac rehabilitation. The authors reported no significant changes in BP following six weeks of IHG training performed at 30% MVC (Table 3). Regarding the feasibility of IRT in patients with IHD, the optimal exercise modality should maximize post-exercise BP reduction while minimizing the magnitude of the BP rise during exercise. The abovementioned preliminary mechanistic research confirms in this population that both the amount of muscle mass engaged during IE and the patient’s training status influence the acute hemodynamic response as well as the magnitude of PEH [65]. However, at present, there are still no data on the tolerability and efficacy of IRT in patients with IHD and adequately powered, medium- and long-term RCTs are needed in this population, Given its greater hemodynamic impact and the requirement for specialized equipment, such as a dynamometer, ILE appears to be better suited for supervised phase II cardiac rehabilitation programs in patients who have already undergone an initial period of exercise training. In contrast, IHG exercise is characterized by a more favorable hemodynamic profile and can be performed using simple, inexpensive devices. These features make IHG particularly attractive for the long-term management of hypertension within unsupervised, home-based, IRT programs, where safety, feasibility, and accessibility are essential considerations.

### 6.2. Peripheral Artery Disease

Correia et al. [66] randomized 102 patients with PAD to IHG or control. The protocol included 4 sets of IHG for 2 min at 30% MVC and a 4-min interval between sets. The study showed that after 8 weeks of home-based exercise, there were significant reductions in diastolic BP and improvement in vascular function in the IHG group compared to control. No changes in systolic BP were observed. The authors attributed the absence of a significant reduction in systolic BP to the fact that the participants were receiving multiple antihypertensive medications, which may have limited the potential for further BP reduction. Interestingly, these findings were comparable to those reported by Gomes et al. [67], who investigated dynamic resistance exercise involving multiple muscle groups despite using exercise sessions that were approximately twice as long as the isometric training protocol used by Correia.

### 6.3. Heart Failure with Preserved Ejection Fraction

Recently, Edwards et al. [68] investigated the feasibility of an IRT protocol, performed at 30% MVC, versus control in patients with heart failure with preserved ejection fraction (HFpEF). The IRT produced significant reductions in resting systolic (−8.52 ± 2.7 mmHg) and diastolic (−4.62 ± 1.8 mmHg) BP, as well as total peripheral resistance (270.4 ± 75.4 dyne s cm^−5^) compared to usual care. Interestingly, although the study was only powered to detect BP differences, authors also found significant positive changes in global longitudinal strain, left atrial reservoir strain, and left ventricular global work efficiency; these preliminary results suggest a potential impact of IRT on central mechanisms that are worthy of further investigation.

## 7. Clinical Implications

The incorporation of IRT into a multimodal strategy that also includes lifestyle modification and pharmacological therapy for the prevention and management of hypertension is particularly appealing in older adults. In this population, long-term resistance training is expected to provide benefits that extend beyond BP reduction, including improvements in skeletal muscle strength and mass, enhanced physical function, quality of life, and favorable metabolic adaptations [69]. To date, three main IRT modalities have been more extensively investigated for their antihypertensive effects: IHG, IWS, and ILE. Although all three modalities have demonstrated BP-lowering efficacy, the majority of the available evidence has focused on IHG for several reasons. First, IHG is a simple, practical, and inexpensive exercise modality that can be performed at home without specialized equipment or advanced physical skills. Second, available evidence indicates that IHG is associated with a favorable hemodynamic profile and appears to be well tolerated, even when performed at relatively high contraction intensities [5,60,61,62]. Both IWS and ILE have also been shown to effectively reduce BP [36,38]; however, their widespread implementation may be limited by practical barriers. ILE requires specialized equipment, incurs higher costs, and generally necessitates supervision by trained personnel, while IWS has been described as physically demanding and challenging [70]. These practical considerations may limit the applicability of IWS and ILE in routine clinical practice, particularly in long-term home-based IRT programs among frail, older adults and patients with cardiovascular disease. Low-intensity IHG (generally performed at 30% MVC) has been consistently demonstrated to induce post-exercise hypotension (PEH) and to produce significant long-term reductions in BP in normotensive, pre-hypertensive, and hypertensive individuals [36]. However, this exercise modality does not appear to elicit PEH in hypertensive patients with underlying cardiovascular diseases, and its long-term effects on BP control in this population remain largely unexplored. In this patient population, further studies are needed to determine the most effective IE protocol for lowering BP. Future research should prioritize IRT modalities and intensities that are capable of inducing a significant PEH response while avoiding excessive left ventricular afterload during the isometric contraction phase.

## 8. Conclusions

Accumulating evidence indicates that IRT is an effective non-pharmacological intervention for lowering resting BP, with reductions that appear clinically meaningful and comparable to those achieved with conventional exercise programmes. The antihypertensive effects of IRT have been consistently demonstrated in normotensive, pre-hypertensive, and hypertensive individuals, appear largely independent of age, sex, and antihypertensive medication use, and have been observed across different IE modalities. Beyond BP control, IRT offers additional advantages, including its simplicity, low cost, time efficiency, and potential to improve muscle strength and physical function, characteristics that make it particularly attractive for older adults and patients who experience difficulty adhering to traditional exercise programmes. Nevertheless, important gaps in knowledge remain. Most available evidence has been derived from relatively small, randomized trials of short duration, predominantly employing IHG, while direct comparisons among different IRT modalities remain scarce. Furthermore, although preliminary studies suggest that carefully prescribed IRT is safe and effective in selected patients with IHD and heart failure with preserved ejection fraction, evidence in cardiovascular populations is still limited. Mechanistic pathways responsible for the antihypertensive effects of IRT—including improvements in endothelial function, autonomic regulation, oxidative stress, arterial baroreflex sensitivity, and myocardial efficiency—also require further elucidation. Future research should focus on identifying the optimal exercise modality, intensity, frequency, and duration for different clinical populations, with particular attention to patients with established cardiovascular disease. In these individuals, exercise protocols should maximize the post-exercise hypotensive response while avoiding excessive left ventricular afterload during isometric contraction. Large, adequately powered RCTs with longer follow-up are warranted to establish standardized IRT prescriptions, evaluate long-term adherence and cardiovascular outcomes, and facilitate the incorporation of IRT into future hypertension and cardiovascular prevention guidelines. Although the low time and equipment requirements of IRT suggest the potential for a cost-effective approach, this remains insufficiently established and should be formally evaluated in future healthcare studies.

## Figures and Tables

**Table 1:** Summary of the results of the cited studies on IRT in normotensive healthy subjects.

Study	Study Design	Population (n)	IRT Modality/Protocol	Main Findings
Goessler et al. [34]	RCT	60	Home-based IHG (4 × 2 min/day at 30% MVC) vs. aerobic vs. control	Both IHG and aerobic exercise reduced office BP, but only aerobic exercise reduced ambulatory BP.
Wiles et al. [35]	RCT	33	Bilateral ILE, high vs. low intensity, 8 weeks	Reduced SBP, DBP and MAP after 8 weeks; none after 4 weeks.
Wiles et al. [36]	RCT	28	Home-based IWS, 4 weeks	Reduced BP and HR.
Hess et al. [37]	RCT	22	IHG at 5% vs. 10% MVC, 6 weeks	Clinically meaningful but non-significant SBP reduction; no DBP reduction.
Baross et al. [38]	RCT	20	ILE at 85% HR peak, 8 weeks	Reduced SBP and MAP; no DBP change.
Badrov et al. [39]	Prospective cohort design	20	Unilateral IHG, 8 weeks	Reduced SBP, DBP, MAP, pulse pressure; increased FMD; no sex differences.
Smart et al. [10]	Meta-analysis	326	Various IRT protocols	BP reduction independent of sex, age, baseline BP, medication or muscle group.
Loaiza-Betancur AF et al. [40]	Systematic review and Meta-analysis	148	ILE, IHG, arm flexion	Reduced SBP, DBP and MAP.
Edwards et al. [41]	Randomised crossover study	21	Cross-over IWS vs. IHG	Both lowered BP; IWS superior for MAP and DBP.
Somani et al. [42]	Prospective interventional cohort study	46	IHG or ILE, 10 weeks	Reduced SBP and pulse pressure; PEH predicted chronic response.

IHG = Isometric handgrip; MVC = Maximal voluntary contraction; BP = Blood pressure; ILE = Isometric leg extension; SBP = Systolic blood pressure; DBP = Diastolic blood pressure; MAP = Mean arterial pressure; IWS = Isometric wall squat; HR = Heart rate; IRT = Isometric resistance training; FMD = Flow-mediated dilation; PEH = Post-exercise hypotension.

**Table 2:** Summary of the results of the cited studies on IRT in hypertensive patients.

Study	Study Design	Population (n)	IRT Modality/Protocol	Main Findings
Carlson et al. [43]	RCT	40	IHG 5% vs. 30% MVC, 8 weeks	30% MVC reduced SBP (−7 mmHg) and MAP (−4 mmHg); no significant changes with 5% MVC.
Palmeira et al. [44]	RCT	63	IHG 30% MVC, 12 weeks	Reduced office SBP; no effect on DBP, ambulatory BP or HRV.
Farah et al. [45]	RCT	72	Home-based vs. supervised IHG vs. control, 12 weeks	Reduced brachial SBP/DBP, central SBP/DBP and MAP; no benefit with home-based training.
Baffour-Awuah et al. [8]	Systematic review and Meta-analysis	415	Predominantly IHG	Reduced SBP, DBP and MAP; greater SBP reduction in unmedicated participants.
Yuan et al. [46]	Systematic review and Meta-analysis	228	RCTs ≥ 8 weeks	Small, non-significant SBP and DBP changes; evidence limited by risk of bias.
Silva et al. [50]	Randomized crossover trial	12	Acute IHG (various protocols)	No significant post-exercise changes in BP, HR or rate-pressure product.
Bocalini et al. [51]	Clinical trial (randomized cross-over)	12	Acute IHG at 30% and 50% MVC	No post-exercise hypotension or significant BP changes.
van Assche et al. [52]	RCT	15	Single bout low-intensity IHG/ILE	Significant post-exercise hypotension during prolonged (6 h) monitoring.
Oliveira et al. [53]	RCT	36	Single bout IHG	Significant post-exercise hypotension with 24-h BP monitoring.
Farah et al. [54]	Systematic review	173	Various protocols	Reduced office BP; acute evidence did not consistently demonstrate PEH.
Wehrmann et al. [55]	Umbrella review	//	Predominantly IHG (4 × 2 min, 3 sessions/week ≥ 8 weeks)	Reduced SBP, DBP and MAP in both normotensive and hypertensive individuals.

IHG = Isometric handgrip; MVC = Maximal voluntary contraction; BP = Blood pressure; ILE = Isometric leg extension; SBP = Systolic blood pressure; DBP = Diastolic blood pressure; MAP = Mean arterial pressure; PEH = Post-exercise hypotension; HRV = Heart rate variability; RCT = Randomized controlled trials; HR = Heart rate; // = Unclear data.

**Table 3:** Summary of the results of the cited studies on IRT in hypertensive patients with ischemic heart disease.

Study	Study Design	Population (n)	Design/Intervention	Main Findings
Caminiti et al. [60]	Case–Control Study	30	ILE at 30% MVC	IRT well tolerated. Higher peak SBP in IHD; increased E/e′, reduced atrial strain, increased wasted work, reduced global work efficiency.
Caminiti et al. [61]	Randomized Pilot Study	48	ILE vs. IHG at 30% MVC in trained IHD	ILE increased SBP and reduced work efficiency; IHG produced no significant BP or echocardiographic changes.
Vitarelli et al. [63]	Cross-over	25	Aerobic vs. bilateral leg extension (20% MVC) vs. control	Both exercise sessions induced post-exercise hypotension; aerobic exercise produced greater effect.
Caminiti et al. [62]	Randomized Pilot Study	54	IHG 30% vs. 70% MVC	70% MVC produced greater post-exercise hypotension than 30% MVC; both intensities had neutral hemodynamic effects.
Gordon et al. [64]	RCT	11	6 weeks IHG at 30% MVC during cardiac rehabilitation	No significant BP changes after training.

IHG = Isometric handgrip; MVC = Maximal voluntary contraction; BP = Blood pressure; ILE = Isometric leg extension; SBP = Systolic blood pressure; IRT = Isometric resistance training; IHD = Ischemic heart disease.

## Data Availability

No new data were created or analyzed in this study. Data sharing is not applicable to this article.

## Abbreviations

The following abbreviations are used in this manuscript:

IHD	Ischemic heart disease
IE	Isometric exercise
BP	Blood pressure
NO	Nitric oxide
FMD	Flow-mediated dilation
IHG	Isometric handgrip
HRV	Heart rate variability
HF	High-frequency
IWS	Isometric wall squat
RCT	Randomized controlled trials
SBP	Systolic blood pressure
DBP	Diastolic blood pressure
PA	Physical activity
MAP	Mean arterial pressure
HR	Heart rate
PEH	Post-exercise hypotension
MVC	Maximal voluntary contraction
ILE	Isometric leg extension
HFpEF	heart failure with preserved ejection fraction
